# A Voice-Annotated Digital Decision Aid to Promote Child Influenza Vaccination: A Feasibility Study

**DOI:** 10.3390/vaccines11030565

**Published:** 2023-03-01

**Authors:** Shih Ying Gun, Aminath Shiwaza Moosa, Chen Wei Poh, Sherryl Lei Ng, Ngiap Chuan Tan

**Affiliations:** 1 SingHealth Polyclinics, Singapore 150167, Singapore; 2 SingHealth Duke-NUS Family Medicine Academic Clinical Programme, Singapore 150167, Singapore; 3 Yong Loo Lin School of Medicine, National University of Singapore (NUS), Singapore 119077, Singapore

**Keywords:** influenza vaccines, parents, child, decision support techniques

## Abstract

(1) Background: Child influenza vaccine uptake is suboptimal due partly to vaccine hesitancy. A voice-annotated digital decision aid, Flu Learning Object (FLO), was developed to facilitate parental decision-making. This study assessed parental views on FLO’s usability and utility and determined its preliminary effectiveness in increasing vaccine intention and uptake; (2) Methods: A single-center mixed method study was conducted in a public primary care clinic in Singapore. Parents of children aged 6 months to 5 years who were unvaccinated in the preceding year were recruited. In-depth interviews explored their views of using FLO. Pre- and post-FLO questionnaires assessed their vaccine intention and perceived usability using the System Usability Scale (SUS); (3) Results: 18 parents were recruited. They became more aware of benefits and potential complications, distinguished influenza from the common cold, and recognized recommendations by National Childhood Immunisation Schedule. FLO addressed parents’ concerns and supported their decision-making process. FLO has good usability with a mean SUS score of 79.3, ranked at approximately the 85th percentile. The usage of FLO significantly increased vaccine intention from 55.6% to 94.4% (*p* = 0.016) with an actual vaccine uptake rate of 50%; (4) Conclusions: Parents generally accepted FLO, which positively influenced their intention to vaccinate their child against influenza.

## 1. Introduction

Influenza is an acute respiratory infection (ARI) caused by influenza viruses which circulate worldwide [1]. Seasonal epidemics occur mainly during winter but may cause more irregular outbreaks throughout the year in tropical regions [1]. Among children under 5 years globally, there were an estimated 109.5 million influenza episodes in the year 2018 [2]. Influenza accounted for 5% of acute lower respiratory infection (ALRI) hospital admissions and 4% of in-hospital ALRI deaths with an estimated 35,000 total deaths from influenza-associated ALRI [2].

With such a high global burden of disease, the World Health Organization (WHO) recommends annual vaccination for children aged between 6 months to 5 years old as a preventive measure [1]. Centers for Disease Control and Prevention (CDC) estimated that influenza vaccination prevented 1.3 million illnesses, 866,000 medical visits, 9000 hospitalizations and 110 deaths among children under 5 years in the United States during the 2019–2020 influenza season [3]. Vaccination also reduced the odds of manifesting fever in breakthrough infections among children by 45% [4]. Despite such benefits of vaccinating children of this age group, the uptake rate is generally suboptimal, such as 44.4% in England and 66.7% in the United States during the 2021–2022 influenza season [5,6].

In tropical Singapore, ARI including influenza is the leading acute medical condition that presents to public primary care clinics, accounting for a quarter of a million attendances in 2021 for a population size of over five million [7,8]. Briefly, 9% to 14% of all pediatric hospitalizations in Singapore are attributed to influenza [9]. Singapore’s National Childhood Immunisation Schedule (NCIS) adopts the WHO’s recommendation with citizens aged 6 months to 5 years old enjoying full subsidy for influenza vaccination [10]. Despite the NCIS’s recommendation, only 7.6% of Singaporean children under 5 years old received an influenza vaccination in 2019 [11,12]. Singapore’s childhood influenza vaccine uptake rate is significantly lower when compared to targets set by other developed nations such as the National Health Service (NHS) England’s target vaccine uptake rate of at least 50% in preschool-aged children [13].

Costs and concerns regarding vaccine efficacy and safety are common barriers to child influenza vaccine uptake as cited by parents [14,15]. Shared decision-making combines such unique parental values and preferences with the best available evidence to guide health decisions including vaccinations [16]. Shared decision-making is an evidence-based approach that increases adult influenza vaccination rates with a pooled odds ratio of 1.96 and may likewise be a strategy to improve child influenza vaccination rates [17]. Decision aids improve patients’ knowledge, reduce their ambivalence and increase their satisfaction with choices made in primary care including vaccinations [18]. Decision aids have also significantly increased confidence in influenza vaccination decisions among healthcare workers while improving child influenza vaccine intention [19,20,21].

Younger and more tech-savvy people (such as parents of young children) prefer digital-based decision aids to paper-based ones as they allow access to more in-depth information beyond the doctor’s consultation room [22,23]. Combining higher interactivity, audiovisual modality and a narrative style, these platforms improve user satisfaction and knowledge recall leading to better-informed decision-making [23,24].

Permission was sought from the creators of the Ottawa Influenza Decision Aid and the decision aid used in Witteman et al.’s randomized controlled trial to adapt them into a parent-targeted decision aid [20,21]. Flu Learning Object (FLO) was thus designed to be an engaging digital-based animated video and incorporated criteria set by International Patient Decision Aid Standards (IPDAS) to qualify as a decision aid [25].

Observing prospective users’ interactions with a decision aid prototype promotes a user-centered design of the tool [26]. Usability and utility testing via surveys and interviews can elicit users’ responses [26]. The International Organization for Standardization (ISO) defines usability as “the extent to which a system, product or service can be used by specified users to achieve specified goals with effectiveness, efficiency and satisfaction in a specified context of use” [27]. Utility denotes the tool’s functionality in providing the features needed by users [28]. Together, usability and utility determine the usefulness of a decision aid [28].

This study primarily aims to assess parental views on the usability and utility of FLO. The secondary objective is to determine FLO’s preliminary effectiveness in increasing vaccine intention and uptake. The authors hypothesized that FLO would demonstrate good usability and utility while positively influencing vaccine intention and uptake.

## 2. Materials and Methods

### 2.1. Study Design Overview

A mixed-methods design was employed to triangulate the findings using quantitative and qualitative methods. Questionnaires were utilized to evaluate FLO’s usability and parental vaccine intention quantitatively. A review of medical records determined actual vaccine uptake. Qualitative assessment was conducted via in-depth interviews with the aid of a topic guide containing questions on usability and utility. 

### 2.2. Study Setting, Sampling and Recruitment

This study was conducted in Eunos Polyclinic, a public primary care clinic in Singapore, from May 2022 to November 2022. The study recruited parents of Singapore citizens or permanent residents aged 6 months to 5 years old. Potential participants were invited to join the study via convenience sampling. Recruitment took place at the end of the child’s clinic consultation or service including routine well-child and developmental screening visits. Written informed consent was then obtained from parents who agreed to complete the questionnaires and interview and have their child’s medical records accessed. Each participant was assigned a unique study identification number to de-identify and ensure confidentiality. This study recruited participants who were at least 21 years old following Singapore’s legal age of consent with no upper age limit. Parents who are not conversant in the English language were excluded as FLO is delivered in English. Those whose children have received influenza vaccines in the preceding year were also excluded as current vaccine intention and uptake were less likely given the recent uptake. This study did not exclude any participant whose child had previously experienced any vaccination-related adverse event, including allergies, as influenza vaccination may not be contraindicated.

### 2.3. Data Collection

The participants attended a single study session conducted via an online video conferencing platform or face-to-face at a suitable date and time as per their individual availability and convenience. Prior to using FLO, parents completed an online questionnaire (Appendix A) that collected their sociodemographic data and assessed their vaccine intention on a 5-point Likert scale (1 = definitely will not vaccinate; 2 = likely will not vaccinate; 3 = undecided; 4 = likely will vaccinate; 5 = definitely will vaccinate). After using FLO, parents completed a second questionnaire (Appendix B) that reassessed their vaccine intention and evaluated parents’ perceived usability of FLO based on the 10-item System Usability Scale (SUS) [29]. SUS was selected as the tool for assessing usability as it is highly valid, reliable and quick to administer [30]. Alternating positive and negative statements in SUS also minimizes extreme response and acquiescence biases [30]. A tool’s usability is considered good when its SUS score corresponds to a rank of at least the 70th percentile [31]. Both the pre- and post-FLO questionnaires were administered using FormSG, which is an end-to-end encrypted online form builder used by Singapore’s public healthcare clusters and government agencies for capturing classified data [32].

In-depth interviews were conducted during the same study session to collect qualitative data on parental views on the usability and utility of FLO. A topic guide (Appendix C) was developed and pilot-tested for reference during the interviews. Each interview lasted between 10 and 30 min and was audio recorded and later transcribed by the study team. The respective children’s medical records on Sunrise Clinical Manager (the electronic medical record system used by SingHealth Polyclinics) were reviewed three months after the study session to determine if there was any actual vaccine uptake after parents used FLO. The participants were remunerated with an SGD20 (approximately USD15) grocery voucher each for their time and contribution to the study.

### 2.4. Data Analysis

Quantitative data were analyzed using IBM^®^ SPSS^®^ Statistics version 27. Participants’ responses on the SUS were analyzed with their sociodemographic variables using a *t*-test, analysis of variance (ANOVA) test and Fisher’s exact test. Pre- and post-FLO intention to vaccinate were compared using the McNemar test.

A framework analysis approach was deployed to identify emerging themes. A coding framework was developed to analyze the qualitative data. After auditing interview transcripts for accuracy, three authors (SYG, ASM, NCT) independently read through the initial three transcripts to closely familiarize themselves with the data. The authors then independently generated a list of initial codes by applying line-by-line coding of the transcripts. Coding discrepancies were resolved via discussion and consensus. Subsequent transcripts were coded by SYG and indexed using NVivo™ version 12 Plus (QSR International). The authors regularly discussed the qualitative data and the codes. SYG interpreted the codes by identifying characteristics and differences between the codes to label the emerging subthemes. Subsequently, the study team reviewed, revised, refined and named the final subthemes. The analysis was undertaken concurrently with data collection to check for data saturation, which was achieved when no new codes emerged and were confirmed by the final three transcripts [33].

## 3. Results

### 3.1. Overview

**Figure 1:**
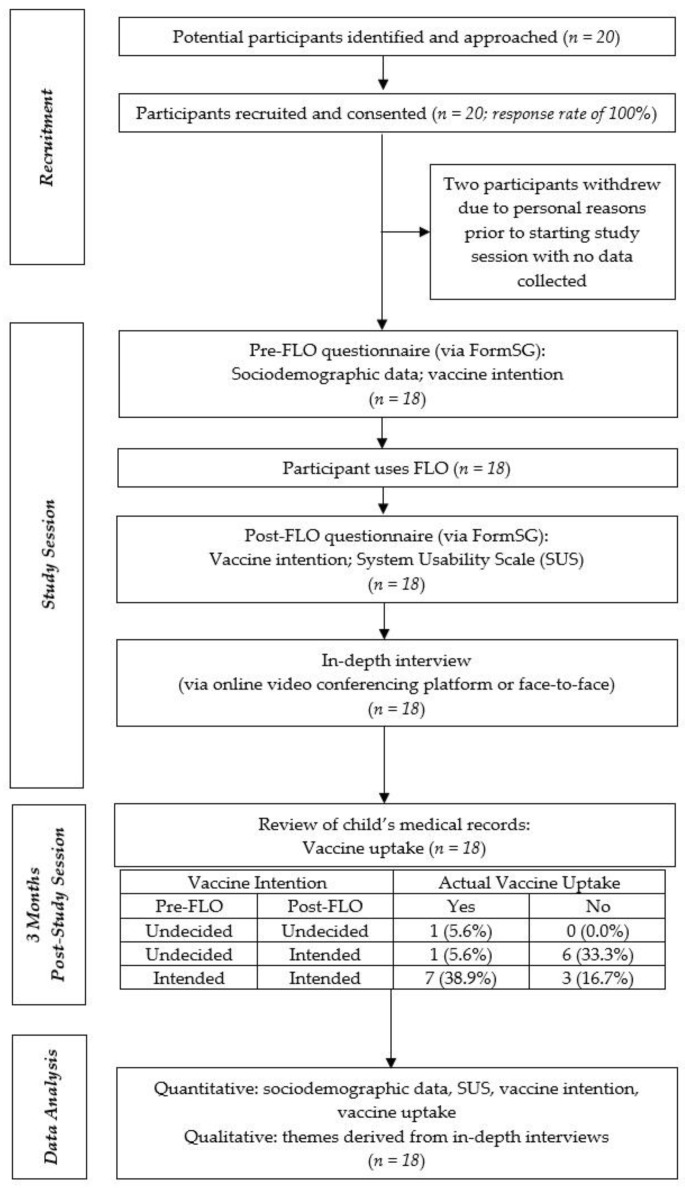
Flowchart of study procedures.

Figure 1 below summarizes the flow of procedures from participant recruitment to the point of data analysis.

### 3.2. Characteristics of Participants

Participants’ sociodemographic characteristics are reported in Table 1.

### 3.3. Usability of FLO on the System Usability Scale (SUS)

**Figure 2:**
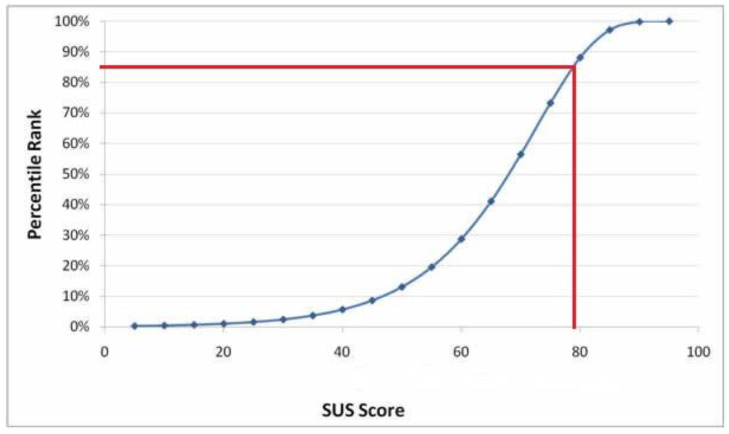
Usability of FLO on the System Usability Scale (SUS).

Participants’ perceived usability of FLO was assessed based on the 10-item System Usability Scale (SUS). FLO has a mean score of 79.3 on the SUS, ranked at approximately 85th percentile, thus showing good usability as demonstrated in Figure 2 [31].

Participants’ responses on the SUS were analyzed with their sociodemographic variables using a *t*-test, an analysis of variance (ANOVA) test and Fisher’s exact test. Within each sociodemographic characteristic category (age, sex, race, highest education level, combined monthly parental income), there is no significant difference in the SUS scores reported (all *p*-values exceed 0.05).

### 3.4. Main Qualitative Findings

The themes and subthemes identified from in-depth interviews with participants are summarized in Table 2.

#### 3.4.1. Usability

##### Clear Wording and Comprehensible Language

FLO’s narrative is generally straightforward with the language used being easy to understand and the fonts used being visibly clear.

*“My English is not really good but the terms they use there are really easy to understand.”*

P2 (Mother of 2-year-and-1-month-old boy)

*“It’s very educational. And it’s very simplified. Easy to understand because it summarized the whole thing. The words (are) also clearly mentioned. It’s quite big and easy to capture because it’s all mainly in black. … So, it catches my attention.”*

P9 (Mother of 7-month-old girl)

A few of the participants felt that the addition of keywords, headers and subtitles may be helpful.

*“I would think that if there are just keywords, it will help me remember better. Maybe just one word of each picture to describe the process. Maybe something like, sort of like “vaccine”, “virus”, “immunity”.”*

P14 (Mother of 6.5-month-old girl)

*“I think subtitles would be helpful too.”*

P6 (Father of 6-month-old boy)

##### Images and Animation Aid Understanding

The illustrations used in FLO aided in parents’ understanding of its contents.

*“I think it was quite well-drawn, the animation images and ya, quite appealing for people to see. I think one of the images that I remember quite clearly was the “A B C” that was talking about school, missing school for children. So, I think when you put this information into images, it does help convey the messages across quite clearly.”*

P17 (Mother of 6-month-old girl)

*“Like with the pie chart, I mean it shows twenty-five percent is substantial so I think that’s easy to understand … I think the use of pictures more than words is great because that will cut through language barriers or age barriers, things like that. And it’s just, you know, easier to digest with pictures.”*

P20 (Mother of 6-month-old boy)

Several parents opined that professional rendering, localized features and improving transition motion may enhance FLO further.

*“I think the features of the visual cues were sometimes a little bit more like Western as compared to like having more Asian kind of features and looks. So, I think maybe just contextualizing it to a more local population might be … So, it’s a little bit more relatable.”*

P8 (Mother of 6-month-old boy)

*“Some way of differentiating each section. Headers will be useful. Colour differences or some animation differences. Maybe the background can be a different colour. So it’s a bit, like a transit to a different topic, subtopic.”*

P17 (Mother of 6-month-old girl)

##### Monotonous and Foreign-Sounding Narrator Can Be Improved

The narrator’s voice is monotonous and foreign, which could potentially be improved by using a more pleasant and locally accented narration.

*“The voice of that narrator is too monotonous. It doesn’t come across as a voice that you trust.”*

P3 (Father of 1-year-and-6-month-old girl)

*“I found the voice-over a little bit unfamiliar, just because of the accent. I’m not sure whether the local population would take to it very well. … I usually think that female voices are a little bit more pleasant.”*

P8 (Mother of 6-month-old boy)

##### Duration Is Acceptable

Parents generally found FLO’s duration to be acceptable.

*“The duration (is) also quite nice, right? It’s like three minutes is good.”*

P1 (Father of 2-year-and-8-month-old boy)

*“Because we are very fast nowadays, we need a short and simple and very understandable message. For a short period of time, everything is compact and all the information is there.”*

P10 (Mother of 1-year-and-7-month-old boy)

#### 3.4.2. Utility

##### Gain Understanding of Influenza and Its Effects

The participants became aware of the possible severe effects of influenza and how it differs from the common cold.

*“It may cause death … wah it’s (a) shocking thing for me”*

P9 (Mother of 7-month-old girl)

*“I think what was really great also was that the video helped me understand the difference between flu and the common cold, which I think is very commonly associated with one another and people think is the same thing.”*

P6 (Father of 6-month-old boy)

##### Raises Awareness of Child Influenza Vaccine

Several participants were unaware of the child influenza vaccine prior to using FLO.

*“For our generation, we don’t do this kind of vaccination, so it’s completely new to us.”*

P1 (Father of 2-year-and-8month-old boy)

*“Actually I’ve only heard about adult flu vaccine … I think a lot of people, a lot of parents, I think they don’t know about this child vaccine.”*

P5 (Father of 6-month-old boy)

##### Recognize Benefits of Vaccination

Apart from protecting against the severe effects of influenza, parents also recognized the potential vaccine benefits in avoiding taking time off work and school and preventing the spread of infection to others.

*“I think the child flu vaccine is kind of helpful to prevent my kid to get a serious illness … he will miss all the classes right because I couldn’t send him to the centre … and then (I) will take off (work) so it will (be) quite a trouble to me. But if you get the vaccine then you might get a milder symptom right. So, the symptoms won’t get so worse until I cannot send my kid to the care centre. So, this is something that I want to avoid. It’s very difficult for the parents right. If both are working parents, so, it’s tough for us to take one week off without proper planning.”*

P18 (Father of 6-month-old boy)

*“(I’m) Scared they will pass it to others, other children in school or will pass (it) to their grandparents.”*

P11 (Mother of 1-year-and-6-month-old girl)

##### Potential Vaccine Side Effects Are Acceptable

Many parents concurred that vaccine side effects were their main concern but were reassured by the information presented by FLO.

*“(FLO) addressed the part on side effects, which I think is very rare (ly mentioned elsewhere). A lot of parents are concerned about side effects. It helps when people know that there’s some mention about side effects from the vaccination.”*

P4 (Father of 4-year-and-9-month-old girl)

*“I see clearly that the side effects are not a big deal for my child but the effect when he gets the flu will be more serious.”*

P2 (Mother of 2-year-and-1-month-old boy)

##### Informs Recommendations by National Childhood Immunisation Schedule (NCIS)

Parents recognized vaccine recommendations in the NCIS along with its financial subsidy, which are motivating factors that encouraged vaccine intention.

*“If it is part of the flow of the vaccination that is recommended, then, yes, I would bring her for it.”*

P14 (Mother of 6.5-month-old girl)

*“This is fully subsidized by (the) government. It’s more encouraging for the people to take. Because some people (are) concerned about the finances. So when they know this is actually fully subsidized by the government, they would love to take for their child’s safety.”*

P10 (Mother of 1-year-and-7-month-old boy)

##### Desire Information on Vaccination Frequency and Access

The participants wanted FLO to include information about vaccination frequency and where they can get the vaccine.

*“I understand in adults, it needs to be taken frequently, so several times, maybe twice a year, if I’m not wrong. So then, I would have a similar question whether that was the case for children also. … How can I register my child for the flu vaccine or how can I get it?”*

P17 (Mother of 6-month-old girl)

*“I would have liked to know more about the seasons, in terms of like how often I need to get the flu vaccine, or how long it would last and things like that. And then like do I just go to the polyclinic to request for it?”*

P19 (Mother of 6.5-month-old boy)

#### 3.4.3. Decision-Making

##### Statistics Quoted Are Useful

Statistics quoted by FLO aided parents in their decision-making process.

*“It’ll make parents want to vaccinate this when they talk about hospitalization and possibility of deaths and gives the figures. I think that’s quite useful.”*

P4 (Father of 4-year-and-9-month-old girl)

*“If I was on the fence, I think I would think about it a bit more just because the statistics really kind of make it quite clear what the risks and benefits are in terms of getting the flu versus if you vaccinate, you get that sixty percent, you know, chance of reducing that risk. … I’m a bit more about probability and statistics. … I’m making my decision based on information and facts.”*

P8 (Mother of 6-month-old boy)

##### Addresses Concerns and Values

FLO appeared helpful in addressing parents’ concerns and clarifying their values in their decision-making process.

*“What I want to know like after the injection (side effects) … Actually it’s more for her health. At the same time the subsidy that actually (makes me) want to go for this injection.”*

P12 (Mother of 4-year-and-2-month-old girl)

*“Think it’s the second or third last slide where it lists out, okay, you need to decide based on your child’s health. Think about impact to child’s health, to other loved ones, financial costs, school or work, et cetera. So that, even if you’re thinking, “okay no”, why don’t you rethink in terms of these aspects, you know, if you’re willing to have them all impacted. I think that is a good reminder to people who maybe think, “okay, maybe I can just risk it, like I don’t need to get vaccinated for every disease out there”.”*

P20 (Mother of 6-month-old boy)

#### 3.4.4. Implementation

##### Implementation Proposals

Parents proposed various suggestions on the ideal timing and setting to view FLO. Suggested timings included before compulsory vaccinations, antenatal and from birth. Suggested settings included clinic, home and school or childcare orientation.

*“I guess maybe at the point when they bring their child for vaccination. … When they’re waiting. Then if the flu (vaccine) is going to be offered to them at the same setting, then they can make a better decision. … Via iPad or maybe on, just on the TV. … So, if it’s shown there in the waiting area, I guess it might encourage some parents and maybe myself to do it at the same setting.”*

P19 (Mother of 6.5-month-old boy)

*“My husband (does) not really support that we should take the vaccines when baby (is) just born. He thinks the baby needs (to grow) big a bit (and) then the brain, the body will develop enough to take the vaccine. He thinks we can just delay the flu vaccine since Singapore is very clean. So, I think it should start from birth then the parents get enough time to make decision. This is a big deal for some parents.”*

P2 (Mother of 2-year-and-1-month-old boy)

*“There is orientation for school. I think schools need to actually promote this video. So when the parents know about this thing, they will love to subscribe it for their child. Cause most of the parents are very busy and (have) no time … They invite parents for the first time (to) go to school, so, parents will know that “oh there’s such thing”. I don’t think a lot of parents out there know about this flu jab.”*

P10 (Mother of 1-year-and-7-month-old boy)

### 3.5. Vaccine Intention and Uptake

**Figure 3:**
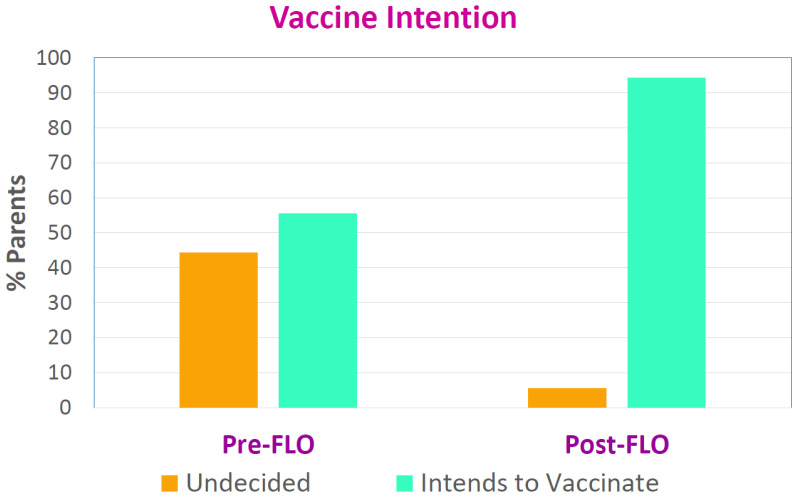
Change in vaccine intention before and after using FLO.

The participants chose either option 3 (undecided), 4 (likely will vaccinate) or 5 (definitely will vaccinate) on a 5-point Likert scale that assessed their vaccine intention. No participants chose option 1 (definitely will not vaccinate) or 2 (likely will not vaccinate). Those who chose either option 4 or 5 were deemed to have a positive intention to vaccinate. The usage of FLO significantly increased vaccine intention from 55.6% to 94.4% (*p* = 0.016) as demonstrated in Figure 3.

A review of the children’s medical records three months after the study session indicated an actual vaccine uptake in 50% of children after their parents used FLO (Figure 1). Briefly, 30% of the parents who indicated positive vaccine intention both before and after using FLO did not vaccinate their children in the end. The only parent who remained undecided even after using FLO, however, ended up vaccinating her child.

## 4. Discussion

Parents generally accepted the FLO prototype, which had significantly influenced their intention to vaccinate their child against influenza. Various design features such as comprehensible language, relevant visual aids, pertinent statistics, and short video duration made FLO highly usable. FLO was useful in addressing parental concerns regarding child influenza vaccination. The participants realized how vaccination could protect their children and other loved ones against the potential severe consequences of influenza while minimizing taking time off work or school. Parents considered the potential vaccine side effects to be acceptable in comparison. Recognizing that the National Childhood Immunisation Schedule (NCIS) recommends and subsidizes the vaccine was also a key motivating factor in encouraging vaccine intention.

Whereas most parents indicated positive vaccine intention after using FLO, only half demonstrated actual uptake. The actual vaccine uptake rate of 50% still remains far higher than the nationwide rate of 7.6% [11,12]. Although, Singapore has not specified any vaccine uptake rate target, the National Health Service (NHS) of England aimed for at least a 50% vaccine uptake in preschool-aged children [13].

Several possible reasons may account for the discrepancy between vaccine intention and actual uptake rates. The medical records reviewed were only for visits to SingHealth Polyclinics. Children who may have received influenza vaccination in other non-SingHealth polyclinics, general practitioner’s clinics or pediatrician’s clinics would not have their vaccination counted for the purpose of this study. A review of the National Electronic Health Record (NEHR) which included National Immunisation Registry (NIR) records would have provided a more accurate finding; however, the Ministry of Health of Singapore had previously precluded using NEHR for research purposes and determined its usage solely for clinical care only.

The timing of the child’s next medical appointment could be another limiting factor. Some of these appointments are well beyond three months from the time of the study session, and parents may potentially opt for convenience in timing their child’s influenza vaccination to coincide with the next medical appointment. Parents may also choose to postpone influenza vaccination so as not to coincide with other childhood vaccinations. Multiple studies have shown that a common barrier to vaccination is having multiple injections during a single visit [34,35,36]. Most parents prefer having no more than two injections per visit due to concerns such as child’s pain and discomfort, compounded vaccination adverse effects, and excessive load on the child’s immune system [34,35,36]. Parents’ acceptance towards receiving more than two injections per visit significantly increases as the child grows older [34]. A longer study duration can perhaps demonstrate if vaccination timings similarly influence actual vaccine uptake amongst study participants.

This study was also conducted in the midst of the COVID-19 pandemic whereby COVID-19 vaccination was available for older children. Discussions were underway of possible vaccination for younger children aged under 5 years old, which is the same age group of the child population of this study. This might perhaps limit parents in vaccinating their children against influenza as they prioritized possible COVID-19 vaccination with dose interval timing considerations.

Ensuring vaccine availability is another important facilitator to vaccine uptake with a lack of access due to vaccine shortage being a barrier [37,38]. Local practices include ordering vaccines in batches to avoid wastage. Parents may intend to vaccinate their child but may not have actual uptake if the vaccine is currently out of stock. Potential strategies to curb the issue include accurate calculations to project vaccine quantity required and implementing an effective vaccine appointment scheduling system.

To the best of the authors’ knowledge, this is the first study in Singapore that evaluates the use of a decision aid on childhood influenza vaccination. The strength of this study lies in its mixed methods approach. Quantitative data on System Usability Scale (SUS) scores is further enriched by contextualized qualitative insights provided by parents during in-depth interviews. A preliminary assessment on FLO’s effectiveness in increasing vaccine intention and uptake also provides an exploratory outlook on its application in actual clinical practice.

FLO is also delivered in English to match the target population’s demographic characteristics. English is the primary language for education and all official communications in Singapore. Nationwide across all ages, the literacy rate is high at 97.1% with 74.3% possessing multi-language literacy and 48.3% speaking English as their primary language at home [39]. In total, 80–90% of those aged between 25 to 50 years old (the typical age range of parents with children under 5 years old) have also attained post-secondary or higher academic qualifications [39]. This is similarly reflected by the highest education level achieved by this study’s participants with the majority possessing university degrees.

The discussion of child influenza vaccination using FLO may likewise encourage discussion and decisions on other childhood vaccinations as parents may have similar concerns for these other vaccines as well. A case for illustration is this study’s P2 who is the mother of a 2-year-and-1-month-old boy who has not received any form of vaccination since birth including those that were mandatory prior to starting formal schooling. The usage of FLO has enabled P2 to better appreciate the importance of vaccines in preventing the severe consequences of communicable diseases and empowered her discussions with her husband. This contributed to P2′s son eventually receiving not only the influenza vaccine but also catching up on all the other childhood vaccines recommended by the NCIS.

This study has its limitations. As discussed earlier, vaccinations performed outside of SingHealth Polyclinics or beyond three months after the study session were not captured. Being a pilot study, its findings cannot be generalized. The participants’ suggestions on further improving the animation features and the narrator’s voice will be considered in further enhancing FLO. Future studies with an adequately powered sample size can assess if the enhanced FLO remains acceptable to parents and increase their vaccine intention with eventual uptake. 

Challenges in adopting and implementing the enhanced FLO in routine clinical practice may include a lack of shared decision-making culture, time constraints and impact on healthcare providers’ workload [40,41]. The adequate training of healthcare providers and systematizing the delivery of FLO to the target population prior to decision-making visits may contribute to successful adoption and implementation [40,41]. The digital nature of FLO can aid such systematic delivery in line with several implementation proposals suggested by participants. Providing an appointment-booking link at the end of FLO can promote vaccine uptake. Linking the measurements of FLO’s usage with the intended outcome data of the vaccine uptake rate can further align key organizational priorities for sustainable integration into routine care [41].

## 5. Conclusions

FLO demonstrates good usability with its high SUS rank of the 85th percentile. The information gained from FLO was helpful in addressing parental concerns about child influenza vaccination and in supporting their decision-making process. The usage of FLO has also significantly increased parental intention in vaccinating their child against influenza from 55.6% to 94.4% with an actual vaccine uptake rate of 50%. The further enhancement of FLO coupled with systematic delivery can contribute to successful implementation in routine care to further improve vaccine uptake.

## Figures and Tables

**Table 1:** Sociodemographic characteristics of study population (*n* = 18).

Characteristics	Number (Percentage)
Age (years)	
21–30	3 (16.7%)
31–40	11 (61.1%)
41–50	4 (22.2%)
Median age in years: 37.0	
Sex	
Male	6 (33.3%)
Female	12 (66.7%)
Race	
Chinese	11 (61.1%)
Malay	4 (22.2%)
Indian/Others	3 (16.7%)
Highest education level	
Up to diploma	5 (27.8%)
University	13 (72.2%)
Combined monthly parental income (SGD)	
≤5000	4 (22.2%)
5001–10,000	5 (27.8%)
10,001–15,000	5 (27.8%)
≥15,001	4 (22.2%)

**Table 2:** Themes and subthemes.

Themes	Description of Themes	Subthemes
Usability	*Ease of using FLO*	Clear wording and comprehensible language Images and animation aid understanding Monotonous and foreign-sounding narrator can be improved Duration is acceptable
Utility	*Benefits derived from using FLO*	Gain understanding of influenza and its effects Raises awareness of child influenza vaccine Recognize benefits of vaccination Potential vaccine side effects are acceptable Informs recommendations by National Childhood Immunisation Schedule (NCIS) Desire information on vaccination frequency and access
Decision-making	*Influence of FLO on vaccine intention*	Statistics quoted are useful Addresses concerns and values
Implementation	*Perceived acceptable time and setting to view FLO*	Implementation proposals

## Data Availability

The data presented in this study are available on request from the corresponding author. The data are not publicly available due to privacy and ethical restrictions.

## Appendix A. Pre-FLO Questionnaire

1. Participant ID No.
2. Gender:
  - Male
  - Female
3. Age:
  - 21–30 years old
  - 31–40 years old
  - 41–50 years old
  - >50 years old
4. Race:
  - Chinese
  - Malay
  - Indian
  - Others
5. Highest education level:
  - Primary school
  - Secondary school
  - Institute of Technical Education (ITE)/Polytechnic/Junior College (JC)
  - University—bachelor’s degree
  - University—postgraduate degree
6. Combined monthly parental income (rounded up to nearest dollar):
  - ≤SGD5000
  - SGD5001–SGD10,000
  - SGD10,001–SGD15,000
  - SGD15,001–SGD20,000
  - ≥ SGD20,001
7. Where do you usually prefer to bring your child to for his/her vaccinations?
  - Polyclinic
  - General practitioner’s clinic (GP clinic)
  - Paediatrician’s clinic
8. Have you ever vaccinated your child against influenza?
  - Never
  - Some years
  - Every year
9. Are you aware that child influenza vaccination is recommended by the National Childhood Immunisation Schedule (NCIS)?
  - Yes
  - No
10. How likely will you be vaccinating your child against influenza this year?
  - 1—Definitely will not vaccinate
  - 2—Likely will not vaccinate
  - 3—Undecided
  - 4—Likely will vaccinate
  - 5—Definitely will vaccinate

## Appendix B. Post-FLO Questionnaire

1. Participant ID No.
2. After using FLO, how likely will you be vaccinating your child against influenza this year?
  - 1—Definitely will not vaccinate
  - 2—Likely will not vaccinate
  - 3—Undecided
  - 4—Likely will vaccinate
  - 5—Definitely will vaccinate
3. Ten statements regarding the usability of FLO are listed below. For each statement, please select the response that best indicates how much you agree or disagree on a scale of 1 to 5 (1 = strongly disagree, 5 = strongly agree).
  - I think that I would like to use FLO frequently.
  - I found FLO unnecessarily complex.
  - I thought FLO was easy to use.
  - I think that I would need assistance to be able to use FLO.
  - I found the various functions in FLO were well integrated.
  - I thought there was too much inconsistency in FLO.
  - I would imagine that most people would learn to use FLO very quickly.
  - I found FLO very cumbersome/awkward to use.
  - I felt very confident using FLO.
  - I needed to learn a lot of things before I could get going with FLO.

## Appendix C. Interview Guide (with Probes)

Q1. Please tell me what you think about the features of FLO.

Content

- How appropriate is the information presented?
- Is there sufficient information provided?

Design

- How do you find the layout of FLO?
- What do you think of the font?
- What do you think of the illustrations?
- Is FLO easy to use and understand?

General

- Are there any features that you particularly like?
- Are there any features that you particularly dislike?
- Are there areas that need changing?

Q2. How useful is FLO in helping you decide whether to vaccinate your child against influenza or not?

- How did FLO affect your decision-making?
- Which section of FLO did you find most useful?
- When do you think is a good time for other parents like yourself to view FLO?

Q3. Do you have any other feedback about FLO?

## References

[1] World Health Organization Influenza (Seasonal). https://www.who.int/news-room/fact-sheets/detail/influenza-.

[2] Wang X., Li Y., O’Brien K.L., Madhi S.A., Widdowson M.-A., Byass P., Omer S.B., Abbas Q., Ali A., Amu A. (2020). Global burden of respiratory infections associated with seasonal influenza in children under 5 years in 2018: A systematic review and modelling study. Lancet Glob. Health.

[3] Centers for Disease Control and Prevention Estimated Influenza Illnesses, Medical Visits, and Hospitalizations Prevented by Vaccination in the United States—2019–2020 Influenza Season. https://www.cdc.gov/flu/about/burden-averted/2019-2020.htm.

[4] Ferdinands J.M., Thompson M.G., Blanton L., Spencer S., Grant L., Fry A.M. (2021). Does influenza vaccination attenuate the severity of breakthrough infections? A narrative review and recommendations for further research. Vaccine.

[5] Public Health England Seasonal Influenza Vaccine Uptake in GP Patients: Winter Season 2021 to 2022. https://assets.publishing.service.gov.uk/government/uploads/system/uploads/attachment_data/file/1128172/GP-patients-flu-annual-report-2021-to-2022-corrected_final.pdf.

[6] Centers for Disease Control and Prevention Flu Vaccination Coverage, United States, 2021–2022 Influenza Season. https://www.cdc.gov/flu/fluvaxview/coverage-2022estimates.htm.

[7] Ministry of Health Singapore Top 4 Conditions of Polyclinic Attendances. https://www.moh.gov.sg/resources-statistics/singapore-health-facts/top-4-conditions-of-polyclinic-attendances.

[8] Singapore Department of Statistics Indicators on Population. https://tablebuilder.singstat.gov.sg/table/TS/M810001.

[9] Ang L.W., Lim C., Lee V.J.M., Ma S., Tiong W.W., Ooi P.L., Lin R.T.P., James L., Cutter J. (2014). Influenza-Associated Hospitalizations, Singapore, 2004–2008 and 2010–2012. Emerg. Infect. Dis..

[10] Ministry of Health Singapore Enhanced Subsidies for Nationally Recommended Vaccinations and Childhood Developmental Screening. https://www.moh.gov.sg/news-highlights/details/enhanced-subsidies-for-nationally-recommended-vaccinations-and-childhood-developmental-screening.

[11] Ministry of Health Singapore Influenza Vaccinations in Children. https://www.moh.gov.sg/news-highlights/details/influenza-vaccinations-in-children.

[12] Singapore Department of Statistics Singapore Residents by Age Group, Ethnic Group and Sex. https://tablebuilder.singstat.gov.sg/table/TS/M810011.

[13] Public Health England Seasonal Influenza Vaccine Uptake in GP Patients: Winter Season 2019 to 2020. https://assets.publishing.service.gov.uk/government/uploads/system/uploads/attachment_data/file/912099/Annual-Report_SeasonalFlu-Vaccine_GPs_2019-20_FINAL_amended.pdf.

[14] Low M.S.F., Tan H., Hartman M., Tam C.C., Hoo C., Lim J., Chiow S., Lee S., Thng R., Cai M. (2017). Parental perceptions of childhood seasonal influenza vaccination in Singapore: A cross-sectional survey. Vaccine.

[15] Kempe A., Saville A.W., Albertin C., Zimet G., Breck A., Helmkamp L., Vangala S., Dickinson L.M., Rand C., Humiston S. (2020). Parental Hesitancy About Routine Childhood and Influenza Vaccinations: A National Survey. Pediatrics.

[16] Elwyn G., Frosch D., Thomson R., Joseph-Williams N., Lloyd A., Kinnersley P., Cording E., Tomson D., Dodd C., Rollnick S. (2012). Shared Decision Making: A Model for Clinical Practice. J. Gen. Intern. Med..

[17] Sanftenberg L., Kuehne F., Anraad C., Jung-Sievers C., Dreischulte T., Gensichen J. (2021). Assessing the impact of shared decision making processes on influenza vaccination rates in adult patients in outpatient care: A systematic review and meta-analysis. Vaccine.

[18] Coronado-Vázquez V., Canet-Fajas C., Delgado-Marroquín M.T., Magallón-Botaya R., Romero-Martín M., Gómez-Salgado J. (2020). Interventions to facilitate shared decision-making using decision aids with patients in Primary Health Care: A systematic review. Medicine.

[19] Vujovich-Dunn C., Kaufman J., King C., Skinner S.R., Wand H., Guy R., Leask J. (2021). A systematic review and meta-analysis of effectiveness of decision aids for vaccination decision-making. Vaccine.

[20] Chambers L.W., Wilson K., Hawken S., Puxty J., Crowe L., Lam P.-P., Farmanova-Haynes E., McNeil S.A., McCarthy A.E. (2012). Impact of the Ottawa Influenza Decision Aid on healthcare personnel’s influenza immunization decision: A randomized trial. J. Hosp. Infect..

[21] Witteman H.O., Chipenda Dansokho S., Exe N., Dupuis A., Provencher T., Zikmund-Fisher B.J. (2015). Risk Communication, Values Clarification, and Vaccination Decisions. Risk Anal..

[22] Politi M.C., Adsul P., Kuzemchak M.D., Zeuner R., Frosch D.L. (2015). Clinicians’ perceptions of digital vs. paper-based decision support interventions: Perceptions of decision support tools. J. Eval. Clin. Pract..

[23] Hoffman A., Volk R.J., Härter M., Li L., Llewellyn-Thomas H., Saarimaki A., Stirling C. Delivering Decision Aids on the Internet. http://ipdas.ohri.ca/IPDAS-Chapter-H.pdf.

[24] De Looper M., Damman O., Smets E., Timmermans D., Van Weert J. (2020). Adapting Online Patient Decision Aids: Effects of Modality and Narration Style on Patients’ Satisfaction, Information Recall and Informed Decision Making. J. Health Commun..

[25] Elwyn G. (2006). Developing a quality criteria framework for patient decision aids: Online international Delphi consensus process. BMJ.

[26] Vaisson G., Provencher T., Dugas M., Trottier M.-È., Chipenda Dansokho S., Colquhoun H., Fagerlin A., Giguere A.M.C., Hakim H., Haslett L. (2021). User Involvement in the Design and Development of Patient Decision Aids and Other Personal Health Tools: A Systematic Review. Med. Decis. Making.

[27] Ergonomics of Human-System Interaction—Part 11: Usability: Definitions and Concepts.

[28] Nielsen Norman Group Usability 101: Introduction to Usability. https://www.nngroup.com/articles/usability-101-introduction-to-usability/.

[29] Brooke J. (1996). A “quick and dirty” usability scale. Usability Evaluation in Industry.

[30] Brooke J. (2013). SUS: A Retrospective. J. Usability Stud..

[31] Sauro J. (2011). A Practical Guide to the System Usability Scale: Background, Benchmarks & Best Practices.

[32] Open Government Products Singapore FormSG. https://form.gov.sg/.

[33] Guest G., Namey E., Chen M. (2020). A simple method to assess and report thematic saturation in qualitative research. PLoS ONE.

[34] Kaaijk P., Kleijne D.E., Knol M.J., Harmsen I.A., Ophorst O.J.A.E., Rots N.Y. (2014). Parents’ attitude toward multiple vaccinations at a single visit with alternative delivery methods. Hum. Vaccines Immunother..

[35] Bakhache P., Rodrigo C., Davie S., Ahuja A., Sudovar B., Crudup T., Rose M. (2013). Health care providers’ and parents’ attitudes toward administration of new infant vaccines--a multinational survey. Eur. J. Pediatr..

[36] Kennedy A., LaVail K., Nowak G., Basket M., Landry S. (2011). Confidence About Vaccines In The United States: Understanding Parents’ Perceptions. Health Aff..

[37] Price T., McColl E., Visram S. (2022). Barriers and facilitators of childhood flu vaccination: The views of parents in North East England. J. Public Health.

[38] Kang G.J., Culp R.K., Abbas K.M. (2017). Facilitators and barriers of parental attitudes and beliefs toward school-located influenza vaccination in the United States: Systematic review. Vaccine.

[39] Singapore Department of Statistics Census of Population 2020 Statistical Release 1: Demographic Characteristics, Education, Language and Religion. https://www.singstat.gov.sg/-/media/files/publications/cop2020/sr1/cop2020sr1.ashx.

[40] Tong W.T., Lee Y.K., Ng C.J., Lee P.Y. (2017). Factors influencing implementation of a patient decision aid in a developing country: An exploratory study. Implement. Sci..

[41] Joseph-Williams N., Abhyankar P., Boland L., Bravo P., Brenner A.T., Brodney S., Coulter A., Giguère A., Hoffman A., Körner M. (2021). What Works in Implementing Patient Decision Aids in Routine Clinical Settings? A Rapid Realist Review and Update from the International Patient Decision Aid Standards Collaboration. Med. Decis. Making.

